# A qualitative exploration of e-cigarette prevention advertisements’ effectiveness among college students in China

**DOI:** 10.18332/tid/189300

**Published:** 2024-06-18

**Authors:** Yu Chen, Shiyu Liu, Yujiang Cai, Rong Gao, Haoyi Liu, Xueying Jiang, Xin Liu, Xinjie Zhao, Zining Wang, Ziyang Chen, Jing Han, Jing Xu

**Affiliations:** 1 School of Journalism and Communication, Peking University, Beijing, China; 2 Global Health Institute, School of Public Health, Xi'an Jiaotong University, Xi'an, China; 3 School of International Studies, Peking University, Beijing, China; 4 Changchun University, Changchun, China; 5 School of International Journalism & Communication, Beijing Foreign Studies University, Beijing, China; 6 Editorial Office of China Medical News, Chinese Medical Association Publishing House, Beijing, China

**Keywords:** e-cigarette prevention, college students, health communication

## Abstract

**INTRODUCTION:**

The rapid growth of e-cigarette usage among youth and young people has emerged as a significant public health concern. It is imperative to initiate effective vaping prevention campaigns and undertake relevant research to address this pressing issue. This research seeks to identify effective video advertisements to deter young people from starting to use e-cigarettes. It aims to offer evidence-based insights and recommendations for creating communication materials and designing messages for youth e-cigarette prevention efforts.

**METHODS:**

College students aged 18–24 years (n=40) participated in focus groups within this qualitative study. After viewing four stimulus videos, participants discussed what they perceived as effective and ineffective video characteristics, as well as suggestions for future videos.

**RESULTS:**

Effective video characteristics included the use of real-life testimonials, displaying specific health hazards, revealing harmful chemical ingredients and the deceptive nature of flavors, and positively perceived effectiveness. Participants generally found that videos with strong visual impact and graphics were more engaging and that approaches using fear and emotion were more effective. Ineffective characteristics included complex and exaggerated information, lack of empathy and irrelevance, insufficiently specific information, extreme and death-themed content, industry messages, as well as preachy tones, animations, metaphors, dull formats, excessive length, and scenes of e-cigarette use.

**CONCLUSIONS:**

Developing anti-e-cigarette campaign materials for youth necessitates target audience-focused qualitative research. This helps in deeply exploring and identifying effective themes and messages, as well as video characteristics and details while avoiding ineffective or even misleading messages and themes from young people’s perspectives outside the United States. Future development of e-cigarette prevention videos for Chinese college students may consider incorporating localized real-life testimonial cases to convey specific harms, including self-efficacy information, and utilizing fear and emotional appeals.

## INTRODUCTION

Electronic cigarettes, commonly referred to as electronic nicotine delivery systems (ENDS) and electronic non-nicotine delivery systems (ENNDS), generate aerosols for user inhalation by heating a liquid solution. These so-called ‘e-liquids’ may or may not contain nicotine (but not tobacco), but they typically include other additives, flavors, and chemicals that may potentially be harmful to human health^1,2^.

The rapid growth of e-cigarette usage can be attributed to aggressive advertising, marketing, and promotional activities conducted via the Internet and social media channels^3-5^. Alarmingly, youth and young adults comprise the majority of e-cigarette users, with use rates experiencing a rapid surge among these groups^6-8^.

From 2015 to 2019, there was a significant increase in the number of e-cigarette users in China, and the country’s adult e-cigarette use rate rose from 1.3% to 1.6%^1^. This rise indicates an addition of approximately 3.35 million new adult e-cigarette users, among which men constituted 3.20 million and women made up 150000. E-cigarettes have also gained popularity among teenagers and young adults in China. The e-cigarette use rate among young adults aged 18–29 years saw a substantial increase from 2.0% in 2015 to 2.7% in 2019. According to a study by China CDC, 14.9% of junior middle school students in China have used e-cigarettes, with a current use rate of 3.0%. Furthermore, based on data published by the China CDC in 2021, the proportion of university students who have used e-cigarettes stands at 10.1%, with the current use rate being 2.5%, both surpassing the national adult tobacco survey results from 2018 ^9-11^.

E-cigarettes, devoid of tobacco, remain deleterious to health, posing a significant safety concern^12^. Notably, their use among children and youth is particularly hazardous. Nicotine has a potent addictiveness, and given that adolescent brains continue to develop until around the age of 25 years, the introduction of electronic nicotine delivery systems to this young population can potentially double their likelihood of becoming traditional cigarette smokers later in life^1^. Increasing evidence suggests that e-cigarettes inflict substantial harm to the physical and mental health of youth during their developmental years. Noteworthy health consequences include lung and brain injuries, overexposure to toxic chemicals, and nicotine addiction^13^. Instituting effective preventive strategies against the significant threats posed by e-cigarettes to global adolescent health is warranted^6,14,15^.

A substantial body of evidence now suggests that anti-tobacco campaigns can effectively prevent adolescent smoking and reduce youth smoking rates, a method that might also apply to e-cigarette use prevention among teens^16^. A study assessing risk communication activities concerning teen e-cigarette use in Virginia noted the effectiveness of anti-e-cigarette campaigns in altering adolescent beliefs^15^. Inspired by the certainty effect in Prospect Theory, a study assessed the cognitive and emotional responses, as well as beliefs, attitudes, and intentions about e-cigarettes, of 536 e-cigarette-susceptible youth after viewing a series of brief messages (eight in total) on the health gains or losses from e-cigarette use. The research found that a loss frame was more effective for those with a high tolerance of uncertainty, while a gain frame maintained a slight advantage for those with low uncertainty tolerance^14^. Some studies, such as Stalgaitis et al.^17^ and Roditis et al.^18^ conducted focus group interviews in the United States to explore adolescents’ reactions to different e-cigarette prevention message content and styles, providing references for the design of prevention advertisements. An experimental investigation into ‘The Real Cost’, e-cigarette prevention advertisements revealed a consistency between perceived and actual effectiveness, both showing a positive effect by increasing risk beliefs and reducing attitudes and intentions towards e-cigarette use^19^.

In recent years, although there have been initiatives to prevent the use of e-cigarettes in some countries, limited research has been carried out on the development of e-cigarette prevention advertisements and messages aimed at youth, which were primarily conducted in the United States. Qualitative research helps to deeply explore personal experiences, feelings, and opinions and discover details and suggestions that are difficult to capture by quantitative testing, providing an important reference for message design. At the same time, we also recognize the advantages of quantitative research, especially experimental research, in testing the causal relationship between message exposure and effects (such as attitude and behavior change). These two research paradigms should be complementary. Given that the public health sector and advertising industry commonly use focus groups for concept and pilot testing, this study aims to provide directional insights for subsequent quantitative evaluation and intervention practice as part of formative research.

As per the United Nations’ definition, youth typically refers to those aged 15–24 years^20^. This study targets college students aged 18–24 years, considering that they are one of the primary user groups of electronic cigarettes. They are also a significant target for e-cigarette marketing. It is crucial for the Chinese government and relevant departments to intervene with targeted efforts, launch effective communication campaigns, and prevent youth and young adults from starting the use of e-cigarettes.

This study is part of a formative research initiative by China’s tobacco control agency to develop a preventative educational campaign addressing adolescent e-cigarette use. The ultimate goal is to establish effective educational activities and promotional materials targeting youth in an effort to prevent e-cigarette use and protect their health.

In alignment with our research objectives, we defined our primary research question as follows: ‘What types of video content do Chinese college students think have the potential to prevent young people from using e-cigarettes?’. We hope to understand the target audience’s feedback and opinions on existing prevention videos and explore insights that can help optimize the design of e-cigarette prevention messages for college students.

## METHODS

### Sampling

Our study utilized purposive sampling. It was conducted from November 2020 to April 2021 in two universities located in Kunming and Beijing. Participants were openly recruited within the campus, with the sample being divided into four groups (10 individuals per group) based on gender: male and female. These cities were chosen with the intent of maximizing sample diversity and facilitating comparison. Beijing, located in Northern China, has made robust strides in tobacco control, implementing smoke-free regulations that align with the World Health Organization’s Framework Convention on Tobacco Control^21^. Public awareness regarding tobacco control in Beijing is comparatively higher. Conversely, Kunming, the capital city of Yunnan province in Southern China; the province is a major contributor to China’s tobacco production and consumption. This has resulted in the economy and public being heavily dependent on tobacco, contributing to lower awareness about tobacco control and relatively high smoking rates. The understanding of harms associated with smoking and e-cigarette use among the youth is notably low^22^.

### Data collection

Semi-structured group interviews were conducted with interview outlines and moderator guides developed in accordance with the research objectives and tested prior to data collection. The research team and the moderators were trained on the methodology and goals of the research project and demonstrated proficiency with interview procedures and techniques. The moderators were graduate students at Peking University, all aged <24 years, a demographic that closely mirrored the interviewees’ age and status. The decision to mostly assign female moderators was made to foster ease and openness in sharing perspectives among research subjects. However, the Kunming male student focus group was given a male moderator, a provision made in light of the relatively high smoking rate among male university students in Kunming. This was done to alleviate any reservations the subjects might have had about sharing their viewpoints, particularly in relation to perceptions, attitudes, and behaviors linked to smoking.

Informed consent was obtained from study participants prior to the commencement of the focus group sessions, ensuring the confidentiality of the discussions. Each focus group interview lasted approximately 40–90 minutes, and each session was recorded. Field notes and data were collected concurrently to uphold the credibility and reliability of the data.

Four different international anti-vaping videos of varying lengths and content from the USA were selected as stimulus videos for this study, including Public Service Announcements and news reports, as detailed in Table 1. The selection of these videos considered diversity in terms of video types (e.g. emotional appeal and rational persuasion), narrative styles (e.g. real-person narrative and animation), and duration (ranging from 30 seconds to over 4 minutes). This selection aimed to cover various representative forms of existing anti-vaping videos to elicit rich feedback on different video types. These materials, obtained online, were translated into Chinese and dubbed with voice-over closely approximating the original. International videos were chosen largely due to the absence of appropriate Chinese material at the time of selection.

**Table 1:** Details of the four stimulus videos used in the study

*Name*	*Duration*	*Main content*
*Who’s Brain Is It Anyway?* Advertisement	30 s	Through narration and visuals, the dangers of e-cigarettes are shown: a plague is spreading. Scientists say it can alter your brain, release dangerous chemicals like formaldehyde into your blood, expose your lungs to chromium, causing irreversible damage. It is not a parasite, not a virus, nor an infection. It is an e-cigarette.
*The Dangers of Vaping* Advertisement	1 min 40 s	With animated visuals, experts explain the marketing tricks of e-cigarettes and their dangers to the human body. E-cigarettes often market themselves as a ‘safe way’ to replace conventional tobacco, with cool designs and a variety of flavors, making them more deceptive and stealthy, thereby attracting teenagers. A medical doctor says e-cigarettes contain a large amount of nicotine, which has harmful effects on the teenage brain. A representative of the Tobacco Termination Project says that quitting e-cigarettes is very difficult, and all e-cigarettes contain harmful components such as diacetyl. The public is urged not to gamble with their health.
*Ontario Teen Suffers Vaping-Related Disease* News	2 min 13 s	US health officials have been trying to understand what causes vaping-related diseases, leading to the death of 28-year-old Kyle Boyd. The teenager’s mother expresses concern about her son’s vaping. Dr Karen Bosma says Kyle’s breathing test still shows significant lung damage, similar to someone who has been smoking for many years. Dr Matthew Stanbrook says that e-cigarettes not only cause various lung diseases but will affect the boy’s life in the long run. Health Minister Patty Hajdu notes the desire to take more robust action to protect people from the effects of vaping and advertising.
*Doctor Issues Stern Warning* News	4 min 34 s	A boy has difficulty breathing due to vaping, and his mother takes him to the emergency room. The doctor discovers that young Adam has lung damage due to vaping. Hundreds of patients nationwide have reported vaping-related illnesses. Thomas Eissenberg, the deputy director of the Center for Tobacco Products Research, says that Adam initially used a nicotine vaping device sold by JUUL, and the immune system reacted to the vaping liquid, causing inflammation. Adam’s lawyer, David Nelman, says that kids like Adam would never smoke or try traditional combustible cigarettes if it wasn’t for JUUL’s marketing practices. After being discharged, Adam still suffers from the impact of vaping, in great pain.

The study opted to use videos rather than still materials because of the video’s unique characteristics. Videos amalgamate visuals, sound effects, and color, providing a more intuitive experience compared to images and texts. Furthermore, in the era of digital media, the target audience primarily gathers information from short videos, video websites, and social media platforms, satisfying the contemporary audience’s viewing needs and aligning with the main content format of digital media platforms.

Before the formal interview, participants in the focus group completed a brief personal information form. This procedure served to further ensure that recruitment fit the study’s conditions. Any participants who did not meet the criteria were politely removed, and alternatives were available if necessary. No participants were found to be non-compliant at the event site. The participants then proceeded with short self-introductions guided by the moderator, followed by the viewing of four e-cigarette prevention videos, each played twice. The interviews unfolded in a sequenced discussion focused on the following aspects: 1) feelings and reactions after watching the four stimulus videos, 2) effective points and reasons, 3) ineffective points or shortcomings and reasons, and 4) recommendations for future videos. The second and third aspects were the focus of this research. In each interview, the moderator repeatedly explained the meaning of ‘effective’ and ‘ineffective’ to the participants. ‘Effective’ refers to any part or whole of the video that may prevent them from starting to use e-cigarettes, indicating positive behavioral potential, and vice versa. The moderator would further ask and explore the reasons why participants considered certain content to be ‘effective’. It should be noted that the assessment of message ‘effectiveness’ in this study is mainly based on the audience’s subjective perceptions and evaluations, which reflect the persuasive potential of the message but are not necessarily equivalent to actual behavior change.

### Patient and public involvement statement

The study participants were not involved in the design, conduct, reporting, or dissemination plans of this research.

### Ethics

The present study received ethical approval from the Institutional Review Board of Peking University (PU IRB), with the review number IRB00001052-20056.

### Participants

According to the research requirements, there were four criteria for participant selection: 1) currently enrolled in universities in Kunming or Beijing; 2) aged 18–24 years; 3) non-users of electronic cigarettes; and 4) willingness to participate in the study and having signed an informed consent form. Details of the recruited participants are given in Table 2.

**Table 2:** Sociodemographic characteristics of the participants

*Characteristics*	*Categories*	*n (%)*
**Gender**	Male	20 (50.0)
	Female	20 (50.0)
	Other	0 (0)
**City**	Beijing	20 (50.0)
	Kunming	20 (50.0)
**Age** (years)	18–24	40 (100)
	Other	0 (0)
**Media used**	Television	14 (35.0)
	Radio	0 (0)
	Newspaper	2 (5.0)
	Subway/bus mobile TV	11 (27.5)
	School promotion platform	25 (62.5)
	Internet	40 (100)
	Other	0 (0)
**E-cigarette use**	No	40 (100)
	Yes	0 (0)

### Data analysis

The audio from the focus group interviews was transcribed, organized, and analyzed, with Nvivo12 software (QSR International Pty Ltd, 2018) for data and materials management. We used a combination of inductive and deductive methods for thematic analysis. First, the research team repeatedly read the interview transcripts and conducted initial coding of the data, identifying recurring topics and concepts. Then, we organized and abstracted the initial codes based on existing literature and research objectives, forming higher level categories and themes. Throughout this process, we constantly compared and revised the themes to ensure they accurately reflected the content of the data. Finally, we selected representative quotes to illustrate and support each theme. Through this series of coding and analysis processes, we ultimately developed a coding matrix encompassing themes, subthemes, and sub-subthemes (Table 3). Key findings were synthesized based on the purpose of the analysis, and representative quotes were selected based on their relevance to the core purpose of our study.

**Table 3:** Thematic descriptions of the four stimulus videos used within the context of this study

*Theme*	*Subtheme*	*Sub-subtheme*
**Effectiveness and reasons**	Theme and message features	Real-life cases
		Positive perceived effects
		Concrete presentation of health hazards and risks
		Chemicals - components and harmful substances
		Revealing the deceptiveness of flavors
		Uncomfortable message
	Video characteristics and style	Short duration
		Details and visual effects
		Fear appeals
		Emotional appeals
**Ineffectiveness, disadvantages, and reasons**	Theme and message features	Low information reception
		Low perceived effectiveness
		Hazards not specific enough
		Industry information/deception
		Death
		Negative emotions
	Video characteristics and style	Metaphor
		Animation
		Dull format
		Preachiness
		Too long duration
		Dubbing issues
		E-cigarette smoking scenes
**Improvements and future video suggestions**	Theme and message features	Real cases
		Specific harms
		Secondhand smoke hazards
		Addictiveness
		Increasing self-efficacy message
		Reasons for starting vaping
		Avoiding preachiness
		Avoiding industry information
	Video characteristics and style	Using real people instead of animation
		Using fear appeals
		Using emotional appeals
		Localization - using Chinese local environment, characters, and language

## RESULTS

A total of 40 participants were involved in this study, all of whom were current university students aged 18–24 years. The cohort was evenly divided between the cities of Beijing and Kunming, with 20 participants from each location, and an equal distribution of males and females. None of the participants was an e-cigarette user. Regular media usage habits of the participants consisted primarily of the Internet (100%), followed by school promotional platforms (62.5%), and television (35%).

Our analysis discerned various prominent themes, subthemes, and sub-subthemes within the data. In accordance with the dimensions utilized in the previous assessment of advertising development and transmission effectiveness, we synthesized key features of effective and ineffective e-cigarette prevention videos, as well as suggestions for future videos, from two perspectives: ‘Theme and message characteristics’ and ‘Video characteristics and style’ (Table 3). The determination of effectiveness and ineffectiveness is based on behavioral potential, specifically on whether the video can deter the viewer from initiating e-cigarette use.

### Effectiveness and underlying causes

*Theme and message characteristics*

Utilizing real-life testimonial cases

The participants generally believed that real-life case studies presented in the videos were effective and served as warnings. The reasons given by the participants included specific information about the diseases and their severe consequences, and authenticity as well:

*‘The content was quite comprehensive as there was a case study, and doctors discussing their experiences with patients. All this combined made it really impactful.’* (Student 1, Female Focus Group, Kunming)

Positive perception of effectiveness

Perception of effectiveness is a judgment of the potential for the message to alter antecedent factors or behavior itself^19^. Indicators include: ‘teaching something new’, ‘causing me to pause and consider’, ‘relevant to me’, ‘powerful’, and ‘persuasive’. In this study, the beneficial points, features, and reasons offered as feedback by participants also include these positive descriptions of perception effectiveness:

*‘This really makes us think twice, like if we don’t fully understand something, we probably shouldn’t just go and try it out.’* (Student 1, Female Focus Group, Kunming)

Health hazards and risks: a detailed presentation

Participants from both Beijing and Kunming believe that a specific, detailed, and intuitive demonstration of the health consequences of e-cigarette use is effective:

*‘The video really makes you see what substances you’re pulling into your lungs when you smoke. It’s quite graphic and the details, like the movement, make you feel like something foreign is invading your body … it’s genuinely scary.’* (Student 4, Male Focus Group, Kunming)

Chemicals

Both male and female participants reported on the efficacy of the chemicals and harmful substances in e-cigarettes:

*‘First off, those ingredients they highlighted are definitely scary, left a strong impression on me. Even if you can’t remember all of them, it’s clear there are a lot of harmful substances, no doubt about that.’* (Student 5, Male Focus Group, Beijing)

Examining the complexity of flavor perception

The message on the intriguing nature of taste disclosed by the male subjects in Beijing and Kunming proved to be effective:

*‘It’s got an educational vibe to it, and one thing that really resonated with me is how they pointed out that e-cigarettes are becoming really stealthy, with all these different flavors, like peach, constantly coming out.’* (Student 8, Male Focus Group, Kunming)

Discomforting message

While ‘discomfort’ appears to be a negative emotion on the surface, participants believe this discomforting message could be effective in preventing the use of e-cigarettes:

*‘Sometimes I think it’s not about leaving a bad impression; it’s that some ads don’t leave any impression at all. It’s better to leave an uncomfortable impression that creates a strong association with something bad. That way, whenever they encounter something bad, they’ll feel that discomfort, and I think that would significantly reduce the likelihood of them engaging in that behavior.’* (Student 10, Male Focus Group, Beijing)

*Video characteristics and style*

Brief duration

The study employed four videos of varying lengths, with participants reporting shorter videos as better suited to their needs:

*‘The first one is pretty short, which is good for ads across platforms. It’s quick to watch, like when you’re just glancing at it on the subway.’* (Student 6, Female Focus Group, Beijing)

Details and visual effects

Participants generally agreed that more visually impactful and cinematic presentations were more engaging, with vivid, suspenseful, and microfilm-like formats yielding better results:

*‘It has a strong visual impact and leaves a deeper impression on the viewer.’* (Student 3, Female Focus Group, Kunming)

Appeal of fear

Participants from the four focus groups all reported being significantly affected by the messages and images related to fear and considered them effective for the prevention of e-cigarette use:

*‘Just thinking about someone’s lungs, all filled up and looking like popcorn, that really scares me!’* (Student 1, Female Focus Group, Kunming)

Emotional appeal

Some participants exhibited strong identification and emotional responses to the themes of familial love and sadness portrayed in the video:

*‘The child ended up in such a state because of vaping, and as a mother, it’s heartbreaking. That’s really moving.’* (Student 6, Male Focus Group, Kunming)

### Limitations and underlying causes

Our observations reveal characteristics and descriptions that contradict the notion of ‘effective points’ in certain respects, also unveiling some novel findings.

*Topic and message features*

Low acceptance of information

The acceptance of information, encompassing both comprehension and credibility, is often employed as an indicator in the development and efficacy studies of anti-smoking message^18,23,24^. Feedback from participants suggested that part of the message presented was too complex and specialized to comprehend effectively, and its over-exaggeration affected its believability, subsequently impairing its impact.

*‘The third video felt a bit over the top … It was hard to believe, that’s the feeling I got.’* (Student 5, Female Focus Group, Beijing)

Reduced perception effect

The participants’ perceived ineffectiveness was primarily exhibited through being unmemorable:

*‘If you can’t remember it naturally, it probably didn’t have much of an effect, no lasting impression.’* (Student 1, Male Focus Group, Kunming)

Insufficient specificity of risks

Participants believed that the presentation of risks was not specific enough, thus reducing its effectiveness:

*‘The filming technique tells you about some dangers, but it feels a bit superficial and not specific enough.’* (Student 7, Female Focus Group, Beijing).

Industry information/industry deception

Our research determined that participants expressed a dislike for industry-related information, perceiving an incongruity between the government’s public statements and the legality of e-cigarettes:

*‘I think they focused too much on the company towards the end. Most of it was about some conflict with the company … I lost track of what they were really trying to say.’* (Student 5, Female Focus Group, Kunming)

Mortality

Participants responded unfavorably to cases involving death, considering them too extreme and merely anecdotal. Such instances reportedly prompted a sense of false security as participants tended to perceive them as unrelated to their individual circumstances:

*‘The third video seemed a bit extreme, just death, like they chose some really extreme cases.’* (Student 2, Female Focus Group, Beijing)

Negative emotions

Some respondents perceived messages that elicit negative emotions, such as unsettling and frightening visuals or data, as a disadvantage. They suggested that uncomfortable content might prompt them to bypass the video. However, participants were often able to provide detailed descriptions of the elements that provoked their discomfort:

*‘You’re supposed to feel scared, and a lot of it is just fear.’* (Student 1, Male Focus Group, Kunming)

*Video characteristics and styles*

The primary shortcomings and criticisms identified by participants included: the use of metaphors, animated visuals, perceived monotony, didactic tone, excessive length, issues with translation and voice-overs, and depictions of e-cigarette use.

Metaphoric expressions

The feedback from participants regarding the use of metaphors was not particularly positive:

*‘The wriggling worms gave it a sci-fi feel, and I just couldn’t take it seriously. I totally missed the point about e-cigarettes.’* (Student 8, Male Focus Group, Kunming)

Animation

Participants reported a disconnect between animation styles and real life, leading to a dislike of the animated format:

*‘Maybe because of my age, I don’t really like ads made in an anime style.’* (Student 8, Female Focus Group, Beijing)

Formal and monotonous aspects

Participants remarked that some of the videos were perceived as dull, presenting informational text in a format akin to a PowerPoint presentation:

*‘The second video just tells us the dangers of vaping … It’s quite monotonous, just someone reading text, making it hard to catch the key points.’* (Student 10, Female Focus Group, Kunming)

Instructional approach

Participants also responded unfavorably to didactic messages, suggesting such approaches often incite rebellious attitudes:

*‘It’s like they’re lecturing you, which I really don’t like.’* (Student 5, Female Focus Group, Beijing)

Excessive duration

The stimulus material for this experiment encompassed two news videos each exceeding two minutes in length, along with an advertisement that ran for over a minute. A common response from the participants pertained to the duration of these materials:

*‘It’s too long, and I couldn’t keep watching till the end.’* (Student 6, Female Focus Group, Beijing)

Issues regarding dubbing

Participants expressed concerns regarding the use of dubbing, suggesting that international films might be better received if only Chinese subtitles were provided, eliminating the need for voice dubbing:

*‘They should have just added Chinese subtitles … especially during the part where the mother was crying; the dubbing was hard for me to accept.’* (Student 1, Male Focus Group, Beijing)

Electronic cigarette use

Participants disclosed a concerning disadvantage that warrants our attention; the portrayal of e-cigarette use can lead to imitation:

*‘In video 4, some extended shots focus on the mouth and nose emitting smoke. I think this might actually make people want to imitate it.’* (Student 8, Male Focus Group, Beijing)

### Improvements and future suggestions for videos

Although participants were not solicited for suggestions on improving the videos, many offered advice on how to enhance the material. Their input, combined with recommendations for future videos, can serve as a valuable reference for the development of new promotional materials in the future.

*Topic and message characteristics*

From the perspective of subject matter and information, participants suggested that future videos could adopt true-case testimonials^1,2^. These could address different topics and central pieces of the message, including concrete harms of e-cigarettes, their addictive nature, and the harms of secondhand smoke^1,2^. Consideration could also be given to including a message on self-efficacy and reasons for starting to vape, while industry information should be avoided. Participants indicated that credible sources are needed and opinion leaders to be trustworthy, but preachy messages should be avoided. They believe young idols could have a certain appeal, but concerns were raised about how these idols might be portrayed as smoking or vaping in film and television, or possibly associated with other negative press:

*‘They should add more actionable message for teenagers, something they can relate to, not just empty preaching.’* (Student 3, Female Focus Group, Beijing)

*Video characteristics and style*

In regard to video features, participants recommended localization of the videos using environment, characters, and language indigenous to China. They further expressed a preference for live-action over animation and advocated for employing elements of fear appeal and emotional appeal:

*‘All the videos we watched are foreign … they don’t address the specific context in China … I found it hard to relate to those scenarios, and the dubbed characters … appearing in news reports … It’s hard to immerse in that setting because the culture is different. So, I think ads featuring Chinese people speaking Mandarin or even dialects would be more effective.’* (Student 1, Female Focus Group, Beijing) *‘If they used the real, raw emotions of a mother’s grief and sorrow, it might have a stronger impact.’* (Student 4, Female Focus Group, Kunming)

## DISCUSSION

Our research explores the characteristics of videos that potentially are an effective deterrent of e-cigarette use among college students^1,2^. From a thematic and message perspective, effective characteristics include the use of real-life cases, the demonstration of specific health risks, the unveiling of harmful chemical components, and the deceptive appeal of flavors. Favorable perceptual effects such as the video and message teaching something new, provoking thought, being relevant, and being persuasive also contribute. In the testing of public service advertisements for smoking and e-cigarette prevention, perceived effectiveness is commonly used as an indicator to evaluate the effect of an advertisement or script. Perceived effectiveness is a judgment of the potential of the message to change antecedents of behavior or behavior itself. In this study, participants reported the effectiveness of the positive perceived effects, aligning with previous research findings. Our study did not find gender differences, both male and female participants acknowledged the effectiveness of the message about the chemical components and harmful substances in e-cigarettes, corresponding to previous studies reporting similar findings where references to chemicals in e-cigarette prevention message, elicited negative associations within the target audience, thereby proving effective^3,4^. In addition, based on participant reports, unsettling messages can effectively prevent the use of e-cigarettes. Being ‘unsettled’ is a negative emotion, but it is effective in preventing negative behavior, a finding that aligns with certain studies on the development of messages encouraging smoking cessation^5,6^.

From the perspective of video style and features, participants generally believed that videos with strong visual impact and expressive imagery are more likely to generate interest. Formats that are vibrant, suspenseful, and similar to microfilms are perceived to be more effective^17^. Comparable findings surfaced in a qualitative study on vaping education initiatives among teenagers in nine US states. Positive feedback was also elicited in our study regarding the use of fear appeals and emotional appeals. There is a wealth of research indicating the effectiveness of fear appeals in various health communication issues, including tobacco control^24^. Emotions of fear have been proven effective in different population groups in altering adverse behaviors and promoting healthy actions. If used appropriately, fear can be a powerful motivator for behavioral change: ‘The most successful advertisements all emphasize compelling, emotion-evoking content’. Short videos were favored by the university student participants, aligning with current trends in new media and social media formats.

Additionally, our study discussed characteristics of ineffective videos that should be avoided in future video production and distribution efforts. From a message perspective, complexity and exaggeration, lack of empathy and relevance, insufficient specificity, and themes related to extreme scenarios and death should be avoided. Participants displayed a dislike for industry-related content, viewing a government stance in combination with legal e-cigarette sales as contradictory. We noted that while participants mentioned discomfort and fear as deterrents to taking in the video content, they were often able to recall disturbing details vividly. This suggests that while these negative feelings led to initial avoidance, they still made a significant impression. This paradox will be further explored in future research.

Regarding video features, it is important to avoid preaching, animations, metaphors, dull formats, excessively long durations, and scenes of e-cigarette use^1,2^. Participants reported that scenes of e-cigarette use could encourage emulation, a point that we must heed and avoid in future video development. Although metaphorical techniques are often employed in anti-smoking adverts, some university student participants responded negatively to such approaches^3,4^. This response aligns with other studies finding critical attitudes towards metaphors among teenagers, potentially due to exaggerated metaphors^5,6^. Participants also raised concerns about voiceovers, suggesting that international clips might be more effective with Chinese subtitles only, rather than voiceovers. Although this point might be based on a misunderstanding of the purpose of the stimulus videos in this research, it still emphasizes the importance of considering voiceover elements that are acceptable to university students and teenagers in future video productions; for instance, adopting a tone that avoids preaching and resonates with their day-to-day lives.

In future videos, participants offered clear and constructive feedback that aligned with effective video characteristics. They expressed a desire for local, real-life testimonials illustrating specific harms. Message pertaining to addiction, secondhand smoke damage, and self-efficacy could appropriately be amplified, while didactic tones and industry-centric perspectives should be avoided. Both fear appeals and emotional appeals can be employed as persuasive strategies. Our study validates the common application of behavior change theories in tobacco control health communication, affirming that emotional and fear appeals, along with self-efficacy, are persuasive message features^25,26^.

The direct feedback and interpretation from participants are invaluable for the development of effective e-cigarette prevention videos tailored for university students. They provide evidence to ensure that e-cigarette prevention campaigns originate from the target audience, rather than solely from the experiences of public health experts, underscoring the importance of this qualitative research.

Lastly, the personal details and findings from our discussions validate that 100% of college students regularly use the internet. As the most active group of internet users, over 98% of these students access the internet multiple times daily across all areas of life^27^. Evidently, with the proliferation of the internet, acquiring health-related messages online has become a significant information source for college students, substantially modifying their lifestyle. Hence, future strategies for promoting anti-electronic cigarette message videos should also prioritize the internet and new media channels to more efficiently reach the target audience.

### Limitations

This study’s limitations include the exclusive use of foreign-produced e-cigarette prevention videos for youth, due to the absence of domestic content in China at the time, potentially affecting the results due to cultural differences. The videos mainly featured younger individuals, possibly leading our college-aged participants not to fully identify with the intended audience, which could have skewed the results. Additionally, this study did not collect information on participants’ combustible cigarette use, considering the potential transitional relationship between smoking and vaping; future research should consider including this factor to more comprehensively understand the responses of different populations to e-cigarette prevention messages. Nevertheless, as a qualitative study, our aim was to explore university students’ perceptions of prevention videos in-depth, and the sample size was sufficient to reach data saturation, providing rich insights for future research and practice. The cross-cultural transferability of the findings might be limited, and future research could explore similar questions in other countries and cultural contexts.

## CONCLUSIONS

Developing anti-e-cigarette campaign materials for youth necessitates target audience-focused qualitative research. This helps in deeply exploring and identifying effective themes and messages, as well as video characteristics and details, while avoiding ineffective or even misleading messages and themes from young people’s perspectives outside the United States. Future development of e-cigarette prevention videos for Chinese college students may consider incorporating localized real-life testimonial cases to convey specific harms, including self-efficacy information, and utilizing fear and emotional appeals.

## Data Availability

The data supporting this research are available from the corresponding author on reasonable request.
